# Tubular Secretory Reserve: A Long-Term Sentinel for Surgical Resilience?

**DOI:** 10.1016/j.ekir.2026.106411

**Published:** 2026-03-02

**Authors:** Zhongjian Xu, Zhongping Cao, Chenyu Zhao

**Affiliations:** 1 Department of Nephrology, The First Affiliated Hospital of Nanjing Medical University, Nanjing, China; 2 Department of China Medical University, School of Pharmacy, The Queen’s University of Belfast Joint College, China Medical University, Shenyang, China; 3 School of Pharmacy, Queen’s University Belfast, Belfast, UK

To the Editor:

We read with great interest the study by Gutiérrez *et al.*,^1^ which demonstrates that a composite score of kidney tubule secretion, measured as a median of 5.5 years before surgery, significantly predicts the risk of acute kidney injury following coronary artery bypass grafting. This finding is pivotal, as it shifts the paradigm of risk stratification from glomerular filtration to tubular secretory capacity, revealing a dimension of renal frailty often masked by preserved estimated glomerular filtration rate.^2^, ^3^, ^4^

However, the substantial time lag between biomarker measurement and the surgical insult warrants critical scrutiny. The authors posit that measuring secretion during “times of relative health” captures intrinsic renal resilience.^1^ Although the predictive robustness (risk ratio 0.49 for the highest quartile) is impressive, this prolonged interval raises questions regarding the longitudinal stability of the secretory phenotype. Unlike the estimated glomerular filtration rate, the trajectory of endogenous secretory markers in aging populations is less well-characterized. Does a high secretion score observed 5 years before reflect a structural nephron mass reserve that buffers against future insults, or does it indicate a sustained metabolic efficiency of proximal tubular mitochondria? Understanding the temporal stability of these markers is crucial before they can be adopted for clinical risk stratification.

Furthermore, the stronger association observed in women is intriguing.^1^ Given the known inaccuracies of creatinine-based estimated glomerular filtration rate in reflecting true renal function in women because of lower muscle mass, it is plausible that the secretion score provides a more physiologically accurate assessment of functional renal mass in this subgroup. This reinforces the need to move beyond creatinine in assessing female kidney health.

Ultimately, this study suggests that tubular health is a distinct clinical entity. If validated, preoperative protocols should evolve to include metrics of tubular reserve, identifying patients who require enhanced renoprotection despite having normal creatinine.

## Disclosure

All the authors declared no competing interests.
